# HER2-Low Breast Cancer: A Review of Epidemiology, Diagnostic Challenges, Targeted Therapies, and Clinical Outcomes

**DOI:** 10.3390/jcm15197559

**Published:** 2026-09-29

**Authors:** Maedeh Mirasheh, Zahra Shahabinia, Mai Abdel Haleem A. Abusalah, Afrooz Mazidimoradi, Leila Allahqoli, Hamid Salehiniya, Do-Youn Lee

**Affiliations:** 1 Department of Midwifery and Reproductive Health, School of Nursing and Midwifery, Tehran University of Medical Sciences, Tehran 1419733171, Iran; 2 Geriatric Health Research Center, Birjand University of Medical Sciences, Birjand 9717853577, Iran; 3 Department of Medical Laboratory Sciences, Faculty of Allied Medical Sciences, Al-Ahliyya Amman University, Amman 19328, Jordan; 4 Health Assistant Department, Shiraz University of Medical Sciences, Shiraz 7193711351, Iran; 5 Faculty of Health Sciences, Cyprus International University, Nicosia 99258, Cyprus; 6 Department of Epidemiology and Biostatistics, School of Health, Social Determinants of Health Research Center, Birjand University of Medical Sciences, Birjand 9717853577, Iran; 7 College of General Education, Kookmin University, Seoul 02707, Republic of Korea

**Keywords:** HER2-low, breast cancer, hormone receptor, trastuzumab deruxtecan, immunohistochemistry

## Abstract

**Background**: Breast cancer is the most common malignancy among women. The HER2-low subgroup represents one of its prevalent subtypes; however, due to its unique characteristics, it presents challenges in diagnosis and treatment, complicating clinical management. This narrative review aims to synthesize evidence regarding the epidemiology, diagnosis, treatment, and prognosis of this subgroup. **Methods**: In this narrative review, following a search of PubMed, Web of Science, Scopus, and Google Scholar databases and the evaluation of 91 studies, findings were synthesized qualitatively and descriptively. Additionally, cohort studies, systematic reviews, and meta-analyses were utilized to explore associations and summarize prognostic trends. **Results**: The prevalence of HER2-low varied between 30% and 65% across different populations, with most studies reporting an association with hormone receptor (HR)-positive status and age over 50 years. Diagnostic challenges primarily stemmed from poor inter-pathologist agreement and laboratory variability. However, emerging technologies such as artificial intelligence (AI) and radiomics have shown promising results in diagnosis. Therapeutically, trastuzumab deruxtecan (T-DXd) demonstrated efficacy with response rates ranging from 21% to 62%; nevertheless, cross-resistance to antibody–drug conjugates (ADCs) and high drug costs remain significant barriers. Patient prognosis and survival, beyond HER2 status, depended on factors such as HR status, Ki-67 index, lymph node involvement, and treatment response. **Conclusions**: HER2-low tumors constitute a distinct, highly prevalent subgroup with specific clinical features, necessitating differentiated diagnostic and therapeutic approaches. Despite the efficacy of T-DXd, obstacles such as intragroup heterogeneity, treatment resistance, high costs, and the lack of standardized diagnostic criteria persist. Therefore, attention to molecular profiling, standardization of diagnostic methods, and the development of cost-effective strategies are essential for equitable access to treatment.

## 1. Introduction

Breast cancer is the most common cancer in women, occurring when control of breast cell growth is lost, leading to the formation of a tumor capable of spreading to other parts of the body [1,2]. In 2022, 2.3 million new cases of breast cancer were diagnosed [3]. According to the International Agency for Research on Cancer (IARC), on average, one in twenty women will develop breast cancer during her lifetime. If current trends continue, the number of new breast cancer cases is projected to reach 3.2 million, and breast cancer-related deaths are expected to reach 1.1 million by 2050 [4,5]. Breast cancer comprises various cellular and molecular subgroups that respond differently to treatment. HER2-low breast cancer, characterized by low levels of human epidermal growth factor receptor 2 protein expression, is diagnosed via immunohistochemistry (IHC) analysis [6,7].

HER2-low tumors account for approximately 30.1% to 65.4% of breast cancers across various populations [8,9,10,11,12,13,14,15,16,17,18,19]. Despite the significant prevalence of HER2-low tumors, prior to the advent of novel therapies, this subgroup was considered part of the HER2-negative category, and treatments similar to those for HER2-negative cancers were applied [19,20,21,22,23,24,25]. Consequently, targeted therapies against HER2, such as T-DXd and sacituzumab govitecan, which are used in HER2-positive tumors, proved beneficial for this group as well [26,27,28,29]. However, treatment response and efficacy in these patients are significantly heterogeneous, influenced by factors such as hormone receptor status, HER2 expression levels, tumor molecular profile, and lymph node status [11,22,28,30,31].

Conversely, diagnosing this subgroup poses challenges. Inter-pathologist agreement in distinguishing HER2-low from other subgroups remains notably poor and frequently discordant [32,33,34]. Furthermore, there is significant variability in HER2-low diagnosis among different laboratories [14]. Additionally, dynamic changes in HER2 status during treatment complicate the selection of optimal therapy [9,30,35,36,37,38,39].

Given the high volume and dispersion of studies in this field, research across various aspects of this subgroup is necessary to provide a comprehensive overview of the current state of knowledge. Therefore, this narrative review aims to synthesize existing evidence to investigate the epidemiology, clinicopathological characteristics, diagnostic challenges, imaging findings, treatment outcomes, and prognosis of HER2-low tumors. This study seeks to identify gaps in current knowledge and lay the groundwork for developing practical strategies to improve disease management.

## 2. Materials and Methods

### 2.1. Study Design

This narrative review was conducted to comprehensively examine existing evidence regarding the epidemiology, clinicopathological characteristics, diagnostic challenges, imaging findings, treatment outcomes, and prognosis of HER2-low tumors.

### 2.2. Search Strategy

A search was performed in the PubMed, Web of Science, and Scopus databases, as well as the Google Scholar search engine, with a time limit of the last 10 years and no language restrictions. The search strategy combined the terms “HER2-low”, “Breast cancer”, “Epidemiology”, “Prevalence”, “Clinicopathological”, “Survival”, “Outcome”, and “Prognosis”. To reduce the possibility of publication bias, searches were conducted across multiple databases, and studies were included regardless of the direction of findings.

### 2.3. Inclusion and Exclusion Criteria

Original studies, including case-control, cohort, and clinical trials investigating the prevalence, clinicopathological characteristics, diagnostic methods, imaging, treatment outcomes, prognosis, and survival in HER2-low tumors, were included. Studies lacking sufficient clinical and epidemiological data, those with small sample sizes, or those that did not specifically address HER2-low were excluded.

### 2.4. Study Selection, Data Extraction, and Synthesis Process

After the initial search and removal of duplicate articles, titles and abstracts were screened based on inclusion criteria, and irrelevant articles were excluded. Full texts were then evaluated, and eligible articles were selected for data extraction. Data extraction was performed qualitatively, and findings related to HER2-low tumors and associated factors were synthesized. Data synthesis was narrative. Due to the nature of this narrative review and the high heterogeneity of the study populations, a meta-analysis was not performed. Findings were reported descriptively, highlighting similarities and contradictions among studies. For conclusions regarding the population and associations, priority was given to valid cohort studies and clinical trials with adequate sample sizes. To ensure methodological rigor and transparency in reporting, this review was conducted following the principles outlined in the Scale for the Assessment of Narrative Review Articles (SANRA). Specifically, we clearly defined the research question, described the search strategy and selection criteria, detailed the data extraction process, and provided a balanced discussion of strengths and limitations. Due to the significant methodological heterogeneity among the included studies—ranging from retrospective cohorts to prospective trials with varying follow-up periods and outcome measures—the application of a single standardized risk-of-bias tool was deemed inappropriate. Instead, we employed a qualitative weighting approach, prioritizing larger cohort studies, systematic reviews, and clinical trials with adequate sample sizes to support our conclusions, while using findings from smaller studies primarily to describe patterns and subgroup variations. Findings from studies with small sample sizes and specific populations were used to further describe patterns and subgroup variations. It is important to emphasize that due to the descriptive and review nature of this study, the findings cannot be used to prove causal relationships, and the reports merely indicate statistical correlations and trends. This narrative review has been reported with full transparency in describing the search steps, selection criteria, and data extraction process.

## 3. Results

In this narrative review, out of 1039 studies identified in the initial search, 91 studies were examined after screening titles, abstracts, and full texts based on inclusion criteria. Given the diversity of studies in terms of methodology, study population, and objectives, findings were organized narratively under the axes of epidemiology, clinicopathological characteristics, diagnostic challenges, imaging findings, treatment outcomes, prognosis, and survival to provide a comprehensive image of existing evidence regarding HER2-low breast cancer.

### 3.1. Definition, Epidemiology, and Prevalence

Review of the studied literature indicated that the prevalence of HER2-low in patients with breast cancer varied between 30.1% and 65.4% across different populations [8,9,10,11,12,13,14,15,16,17,18,19]. In large-sample studies, the prevalence of HER2-low was reported as 60.5% of 59,907 patients [14], 65.4% of 38,773 patients with stage 0–3 HER2-negative breast cancer [15], 44.9% of 14,642 patients [16], 47.6% of 8026 invasive cancer samples [17], 47.72% of 3376 patients [18], and 57.5% of 597 patients [19]. In patients with metastatic breast cancer, the prevalence of HER2-low in metaplastic breast cancer (MCB) patients was 59% [19]. It should be noted that the wide range of reported prevalence suggests significant statistical variance between studies, largely due to differences in sample sizes (ranging from 597 to over 59,000 patients), different geographic populations, heterogeneous diagnostic criteria, and unbalanced distribution of clinical subgroups. Therefore, due to the impossibility of statistical weighting in this narrative review, the reporting of these raw figures is merely descriptive and should not be considered as definitive estimates [8,9,10,11,12,13,14,15,16,17,18,19]. Additionally, another study reported the highest rate of brain metastasis in the HR-negative HER2-low group [40]. In these studies, HER2-low was predominantly defined as IHC 1+ or IHC 2+ tumors with negative FISH/ISH [19,20,21,22,23,24,25]. One study emphasized the simultaneous reporting of IHC and ISH scores along with a specific classification for HER2-low to identify HER2-low patients who could benefit from ADC-based therapies [41]. Subgroup distribution in most reports favored IHC 1+; for example, in one study of 597 patients, 207 (34.7%) had IHC 1+ and 136 (22.8%) had IHC 2+/ISH-negative [19]. In another study, among 963 patients with HER2-low status, 70.6% were IHC 1+ and 29.4% were IHC 2+/ISH [42]. Similarly, in a study with 203 patients, 72.4% were in the IHC 1+ group, and 27.6% were in the IHC 2+/FISH-negative group [25].

Demographically, in most reviewed studies, the age of HER2-low patients was around 50 to 65 years [10,11,20,26,42,43]. Most studies found no significant difference in age between groups [44,45,46], whereas one study reported that HER2-low patients were significantly older than HER2-zero patients [10]. Furthermore, the prevalence of HER2-low was more frequently reported post-menopause in most studies [10,11,18,27,46,47,48,49,50,51], although some studies found no significant difference in menopausal status between groups [44,45,52,53]. Additionally, HER2-low was more prevalent in white patients in some studies [26,39,42,48]. However, a few studies found no significant difference in race, smoking status, body mass index, or family history of breast cancer between HER2-low and HER2-zero groups [44,45,46]. Moreover, the prevalence of HER2-low was higher in HR-positive breast cancer patients compared to HR-negative patients [15]. Overall, HER2-low constitutes a significant portion of breast cancer patients, but reported prevalence varies across populations and depends on the criteria used for HER2 classification. The definitions of HER2 subgroups based on the reviewed studies are summarized in Table 1.

### 3.2. Clinicopathological Characteristics

An examination of the clinicopathological characteristics of included studies revealed that patients with HER2-low breast cancer differed significantly from other HER2 subgroups (HER2-zero and HER2-positive) in terms of hormone receptor status, tumor grade, lymph node involvement, and Ki-67 index [11,12,13,16,18,20,45,46,59,60,61,62]. The most common finding was hormone receptor status, with HR, PR, and ER positivity significantly higher in HER2-low patients compared to HER2-zero [15,16,18,19,20,63]. Additionally, in several investigations, HER2-low patients had lower histological tumor grades and lower Ki-67 proliferation indices [10,15,18,50,52,60,61]. However, some other studies associated HER2-low with higher tumor grade, Ki-67, lymph node metastasis, and increased stromal tumor-infiltrating lymphocytes (sTILs) [10,12,13,60,64].

Compared to the HER2-positive group, HER2-low patients showed significantly higher rates of age over 50, tumor size greater than 20 mm, and Ki-67 ≤ 14% [15,16]. Generally, HER2-low patients represent a subgroup with distinct clinicopathological characteristics, marked by high hormone receptor expression and lower tumor grade and proliferation; however, these features were observed differently in some studies and populations.

### 3.3. Diagnostic Challenges

One of the most important diagnostic challenges in breast cancer is the precise differentiation of HER2-low from HER2-ultralow. Studies have indicated that multiple factors, such as distinguishing membranous staining from cytoplasmic staining, peripheral staining (edge artifacts), and the 10% threshold for HER2-ultralow, are primary diagnostic challenges [32,41]. Furthermore, pre-laboratory steps, such as reducing cold ischemia time to less than 1 h, fixation in neutral buffered formalin for 6 to 72 h, regular tissue processing, and the use of external controls, are fundamental requirements. Additionally, tissue fixation, antigen retrieval techniques, enzyme-chromogen reaction time, antibody incubation temperature, and substrate/chromogen concentration affect the intensity of IHC staining [41]. In addition, in many patients diagnosed with HER2-low, central review with IHC 0 (with or without staining) revealed they were actually HER2-ultralow [26]. These variations in antibody clones and quality control methods are summarized in Table 2.

Moreover, tumor biological features complicate diagnosis. Intratumoral heterogeneity and the semi-quantitative, subjective nature of evaluation are factors contributing to disagreements among pathologists. To reduce diagnostic errors, suggestions include using high-power microscopy (40×) to distinguish between IHC 0 and IHC 1+, using control groups with a wide spectrum of IHC 1+ expression, and performing reflex ISH in cases of ambiguity between IHC 1+ and IHC 2+ or in cases where complete membrane staining at 3+ intensity is observed in less than 10% of tumor cells. Despite this, there is disagreement among specialists regarding the relationship between natural epithelium staining in generating false positives and the definition of HER2-ultralow due to limited evidence and unclear guidelines; most prefer using specific IHC scores for clarity [41]. Consistent with these findings, in one study, complete agreement among seven pathologists was achieved in only 33.3% of cases (in HER2 1+ and HER2 2+ groups), with the greatest disagreement (53.3%) concerning the distinction between HER2-null and HER2-ultralow groups, where null cases were often reclassified as ultralow. These findings indicate greater difficulty in diagnosing HER2-ultralow compared to other groups for pathologists [32].

Another diagnostic challenge is the variability in HER2-low rates between laboratories and across antibody clones. For instance, in one study across 29 laboratories, among HER2-negative cases, the range of HER2-low rates varied from 39.8% to 95%. Furthermore, based on antibody clones, DG44 showed a mean sensitivity of 86.3%, while 4B5-Ultraview showed 59.9% [14]. In another study, agreement was higher in biopsy samples than in resection samples, indicating the impact of sample type on HER2 assessment accuracy [33].

In recent years, the use of artificial intelligence, computational pathology, and certain software has gained attention to reduce variability among pathologists [34,41]. Evaluation of the HER2 APP, CE2797 (VP APP; Visiopharm, Hoersholm, Denmark) showed that the highest instances of disagreement occurred between scores 0 and 1+, as well as 1+ and 2+, near diagnostic thresholds. Additionally, in cases where specialists disagreed on a score of 2+, VP APP downgraded their scores to 1+, all of which were FISH-negative, indicating over-scoring by visual assessment [34]. Furthermore, despite incomplete consensus on AI’s ability to improve the diagnosis of HER2 0 and HER2 1+, most specialists agreed that computational pathology could be used in the future as a complementary tool for HER2 scoring [41]. It is important to recognize that the assessment of the HER2-ultralow phenotype remains technically and diagnostically challenging due to the extremely low level of protein expression and the potential impact of pre-analytical, analytical, and interpretative factors. Although HER2-ultralow has been increasingly incorporated into the current HER2 classification framework, its accurate identification remains difficult, particularly in distinguishing cases with faint or barely perceptible membranous staining from HER2 IHC 0 cases. Variability in staining procedures, scoring criteria, and interobserver interpretation may contribute to differences in the reported prevalence of HER2-ultralow tumors across studies [65,66,67]. Furthermore, nonspecific or ambiguous staining patterns may increase the risk of misclassification, highlighting the need for standardized assessment protocols, validated assays, and appropriate quality control measures. Consequently, differences in laboratory practices and interpretation approaches should be considered when comparing diagnostic outcomes and clinical data across studies [65,66,68].

**Table 2:** Reported antibody clones and quality control methods in reviewed studies.

Antibody Clone/Kit Reported	Quality Assessment Method/Quality Control	References
4B5 (Ventana/Roche/PATHWAY), including VENTANA 4B5, PATHWAY 4B5, Roche 4B5, rabbit monoclonal 4B5, and anti-HER2/neu (4B5)	Simply stating that IHC should be performed according to ASCO/CAP guidelines and reviewed by experienced pathologists. Use of control cells with a spectrum of expression (Dynamic Range cell lines), IHC calibrators (peptide-coated microbeads), and artificial intelligence software (Qualitopix; Visiopharm) to measure the limit of detection (LOD) and evaluate laboratory performance. Use of positive controls (endometrial tissue and breast carcinoma) and internal control (normal breast tissue) and review at 40× magnification. Use of digital image analysis software (VP APP, and Visiopharm) and comparison with the consensus opinion of 16 pathologists. Special training of pathologists for HER2-low and HER2-ultralow scoring.	[13,14,21,22,23,26,32,33,34,37,52,59,62]
DG44 (Dako; Glostrup, Denmark)	Use of control cells and IHC calibrators to measure limit of detection (LOD) and evaluate laboratory performance.	[14]
A0485 (Dako)	Use of control cells and IHC calibrators to measure limit of detection (LOD) and evaluate laboratory performance.	[14]
SP3	Use of control cells and IHC calibrators to measure limit of detection (LOD) and evaluate laboratory performance.	[14]
A485 polyclonal antibody (Dako), HER2 IQISH pharmDx kit (for ISH)	Report on the revision of HER2 scoring based on the 2018 ASCO/CAP guidelines.	[69]

Of the reviewed studies, 14 reported the antibody clones used, which are summarized in this table. Clone 4B5 (Ventana/Roche) was the most commonly used antibody (13 studies). Clones DG44, A0485, SP3, and A485 were each reported in only one study. The methods mentioned in some studies (such as control cells, IHC calibrators, AI software, and pathologist training) are related to improving the accuracy of interpretation and not to quality control of the antibody itself. All antibodies discussed are commercial products approved with valid quality standards. Most studies also performed HER2 assays according to ASCO/CAP guidelines using standard positive and negative controls.

### 3.4. Imaging Features and Magnetic Resonance Imaging (MRI)-Based Prediction Models

#### 3.4.1. MRI Morphological Features

Various studies have investigated the imaging features of HER2-low tumors to differentiate this subgroup from HER2-zero and HER2-positive groups. In a retrospective study of 191 patients (139 HER2-low), MRI showed a mean tumor diameter of 20 mm, with 73% being single lesions and 27% multifocal. Morphologically, 74% of HER2-low tumors appeared as masses and 26% as non-mass enhancement (NME). Ill-defined margins were seen in 92% of cases, and an irregular shape in 74% of cases. A washout pattern in DCE kinetic curves was observed in 77% of cases, and peritumoral edema in 54% of cases. Heterogeneous contrast enhancement was present in 87% of cases, with a mean apparent diffusion coefficient (ADC) of 0.89 × 10^−3^ mm^2^/s. Among the examined variables, only ADC showed a significant difference in the HER2-low group compared to the HER2-positive group; no significant differences were observed in other features such as lesion number, morphology, edema, DCE curve, enhancement pattern, and BI-RADS. Additionally, multifocality, non-mass enhancement, peritumoral edema, and washout pattern were significantly lower in the HER2-low group than in the HER2-positive group [47]. Other studies also found significant differences between HER2-low and other HER2 groups in MRI. Compared to HER2-positive, the HER2-low group showed mild or no contrast enhancement, denser fibroglandular tissue, and smaller long and short axis diameters [70]. In a larger study comparing 483 HER2-low cases with 149 HER2-zero cases, differences were observed in tumor morphology (74.5% irregular in HER2-low vs. 53.7% in HER2-zero), initial enhancement rate (0.737 vs. 0.613), and minimum ADC values (0.987 vs. 1.089) [71]. A summary of MRI morphological features across HER2 subgroups is presented in Table 3.

#### 3.4.2. Radiomics Models

Radiomics refers to the extraction and quantitative analysis of imaging features from medical images, allowing the identification of subtle patterns that are difficult to detect or quantify with the human eye, such as HER2 status, in a non-invasive manner [8,70,72,74,75]. Radiomics-based models have shown promising performance in predicting HER2 status. In a study of 220 patients (82 HER2-low), it was shown that tumors in this group had higher intratumoral Kep (rate constant) and Ktrans (volume transfer constant) and a lower TME (tumor microenvironment) compared to both HER2-zero and HER2-positive groups [70]. In another study of 118 patients (78 HER2-low and 40 HER2-zero), TIC pattern III in DCE-MRI was identified as the strongest independent factor for identifying HER2-low tumors. Additionally, radiomics models differentiated these tumors from the HER2-zero group with relatively good accuracy, with the best diagnostic performance observed when a combined nomogram (including TIC and radiomics) was used, indicating the effective role of non-invasive diagnostic methods in diagnosing HER2-low [72]. Furthermore, the Habitat model, based on the XGBoost algorithm and using radiomic features extracted from MRI, was able to diagnose HER2-low tumors in three different centers with high accuracy (AUC between 0.844 and 0.877). Additionally, signal homogeneity, possibly related to dense vascular tissue, was identified as the most important feature for predicting HER2-low and is characteristic of these tumors [8]. The performance of radiomics and machine learning models is also summarized in Table 2.

#### 3.4.3. Machine Learning Models and Novel Biomarkers

Machine learning models have also been used to distinguish HER2-low from HER2-positive tumors. One model was constructed using three MRI features: multifocal lesions, peritumoral edema, and tumor margin. Decision Tree and SVM models performed best, with a training AUC of 0.79; however, performance decreased in the test set (AUC 0.67–0.69). Other models such as KNN and ANN showed lower performance (AUC 0.64 and 0.61, respectively) [47]. These models indicated that HER2-low tumors exhibit more complex and heterogeneous patterns on MRI. Tumors classified as HER2-low by these models also showed distinct gene expression patterns, particularly in immune-related pathways, and provided prognostic information beyond standard pathological classification [49]. In addition, the intratumoral and peritumoral heterogeneity score (ITH) was significantly higher in HER2-low compared with HER2-zero and lower than in HER2-positive tumors. Incorporating this score into prediction models significantly improved the accuracy of HER2-low diagnosis [73]. A summary of these models and their performance is presented in Table 3.

Although machine learning and radiomics models have shown promising results, several important statistical limitations must be considered. The drop in performance from training to test sets (e.g., SVM: AUC 0.79 → 0.67) indicates a risk of overfitting and questions the generalizability of these models to new populations [47]. Class imbalance is also common in the reviewed studies, with HER2-low cases substantially outnumbering HER2-zero cases, which may artificially inflate AUC values [49]. With the exception of the Habitat model (based on XGBoost), which underwent external validation across three centers, most other models lack robust external validation and require confirmation in larger prospective studies [8]. Until these limitations are addressed, clinical application of these models should be approached with caution.

### 3.5. Treatment Outcomes in HER2-Low Tumors

#### 3.5.1. Antibody–Drug Conjugates (ADCs) and Targeted Therapies

##### Trastuzumab Deruxtecan (T-DXd) and Its Combination with Other Drugs

Traditional anti-HER2 therapies such as trastuzumab require high receptor density for efficacy and are therefore ineffective in HER2-low and HER2-ultralow tumors [76]. This limitation has prompted the investigation of novel therapeutic strategies such as ADCs with different mechanisms of action. The efficacy of T-DXd in HER2-low patients has been investigated in various studies. The combination of Durvalumab and T-DXd in first-line HER2-low and HER2-negative patients resulted in an objective response rate (ORR) of 62.1% and a median progression-free survival (PFS) of 12.6 months [26]. In another study involving 70 HER2-low patients, the mean PFS was 5 months, and the ORR was 21.4%. Patients with IHC 2+ had longer PFS compared to IHC 1+ (5 months vs. 3 months) and were identified as an independent predictive factor for improved PFS [27]. In a multicenter study in China involving 46 HER2-low patients, the mean PFS was 8.8 months and the ORR was 19.5%, attributed to more advanced disease conditions and a higher prevalence of brain metastasis [11]. In another review of 29 HER2-low patients, the mean PFS was 10.51 months, and the ORR was 37.93% [28]. Furthermore, a study on 30 HER2-low/zero patients with brain metastasis showed that T-DXd could induce acceptable intracranial responses. In this study, HER2-positive patients had better PFS than HER2-negative patients (10 months vs. 3 months; *p* < 0.001) [29].

In a study of 101 patients, the combination of T-DXd with other drugs in HER2-low patients was examined. The combination of T-DXd with anastrozole yielded the highest ORR (71.4%) and PFS (13.4 months). In the combination of T-DXd with capivasertib, patients with PIK3CA/AKT1/PTEN mutations had higher responses (76.9% vs. 52.4%), and interstitial lung disease (ILD) occurred in 0 to 25% of patients in different modules [77]. In a preclinical study, the combination of this drug with capivasertib in HER2-low cell lines and xenograft models showed that this combination induced increased cell death and cell cycle arrest by inhibiting the PI3K pathway and causing DNA damage. This combination yielded better responses in animal models than monotherapy, with a low risk of hematotoxicity reported [24]. In another study across 25 centers in France involving 179 patients with metastatic HER2-low cancer, it was shown that HER2 status can change between primary and metastatic tumors; specifically, 19% of patients who were initially HER2-zero converted to HER2-low upon metastasis. Based on the patient’s hormone receptor status, the sequence of using two drugs, T-DXd and SG (sacituzumab govitecan), differed; in HR-negative patients, SG was mostly used first, followed by T-DXd, whereas in HR-positive patients, the order was reversed. The mean time to progression-free survival for the second treatment in the entire population was 2.7 months, and if T-DXd was used as the second treatment, this time was longer than with SG (3.1 months). Furthermore, the response rate to the first treatment (35.4%) was significantly higher than to the second treatment (19%). About half of the patients who had a relative response to the first treatment showed primary resistance to the second treatment, indicating the presence of cross-resistance between these drugs [30].

##### Other ADCs and Targeted Therapies

In a study of 46 HER2-low patients, treatment with disitamab vedotin (RC48-ADC) resulted in a mean PFS significantly lower than that of the HER2-positive group (3.45 months vs. 5.26 months) [78]. In another study of 54 HER2-low patients treated with pegylated liposomal doxorubicin (PLD), the mean PFS was 2.6 months, showing no significant difference from HER2-zero patients (3.1 months), and HER2-low was not an independent factor for rwPFS [58]. In another study of 50 patients with HER2-low metastatic breast cancer (MBC) treated with RC48, the mean PFS was 4.28 months, the ORR was 26%, and the DCR was 64%. After discontinuing RC48, the most common subsequent treatments were single-agent chemotherapy (40%), taxane/platinum regimens (26.7%), and ADCs such as SG (13.3%), T-DXd (10%), and SKB264 (6.7%) [79]. Secondary resistance to ADCs is one of the therapeutic challenges; the secondary response rate to the second ADC (19%) decreased compared to the first ADC (35.4%), and 49.4% of patients with secondary resistance to the first ADC showed primary resistance to the second ADC, indicating relative cross-resistance [80]. It should be noted that patients receiving T-DXd and RC48 differed in the number of prior lines of therapy, which limits direct comparison of the ORR between these agents. Response to treatment, especially ADCs, varies based on HR, HER2 expression levels, and molecular profile [11,22,28,31].

#### 3.5.2. Endocrine Therapy and CDK4/6 Inhibitors

Various studies have investigated the effect of CDK4/6 inhibitors in HER2-low patients. In a study of 309 patients (122 HER2-low), the mean PFS in HER2-low was 23.9 months and in HER2-zero was 25.2 months, with no significant difference [46]. Another study of 219 HER2-low patients treated in the first line with CDK4/6 inhibitors and aromatase inhibitors (AI) compared to AI alone found no significant difference in PFS between HER2-low and HER2-zero. The combination therapy was associated with reduced risk compared to general HER2-low vs. HER2-zero, but the interaction test was not significant [81]. These consistent findings suggest that the prognostic impact of CDK4/6 inhibitors is robust, regardless of minor discrepancies in HER2-low classification potentially arising from antibody specificity or storage conditions. In the PALOMA-2 study of 666 patients (513 patients with HER2-low), adding palbociclib to letrozole in the HER2-low group significantly increased PFS (mean 24.8 months vs. 13.8 months), but no significant difference was observed in the HER2-zero group [82]. In the PALOMA-3 study of 520 patients (367 HER2-low), adding palbociclib to fulvestrant in the HER2-low group significantly increased PFS and improved the clinical benefit rate (CBR). In the HER2-zero group, PFS was significant in the primary analysis but not confirmed in sensitivity analyses, and the ORR, CBR, and OS showed no difference [82].

#### 3.5.3. Chemotherapy

Various studies have shown that HER2-low tumors have a lower pathological complete response (pCR) rate to neoadjuvant chemotherapy compared to HER2-zero. In a study of 103 patients (55 HER2-low) receiving at least two cycles of chemotherapy (mean of five cycles), the short-term efficacy ORR in the HER2-low group (especially in the HR-negative subgroup) was significantly higher than in HER2-zero (56.4% vs. 27.1%; *p* = 0.003); however, PFS did not change significantly, and side effects of this chemotherapy included reversible grade 3-4 neutropenia and anemia, unrelated to treatment-related mortality (approximately 15.53%) [83]. In the high-genomic-risk subgroup (Oncotype DX RS ≥ 26), 78.4% of HER2-zero patients and 78.9% of HER2-low patients underwent neoadjuvant chemotherapy, and better survival outcomes were observed in the HER2-low group compared to HER2-zero [84]. In another study of 252 luminal patients undergoing neoadjuvant chemotherapy, 230 patients received anthracycline followed by taxane, 1.2% received carboplatin and taxane, 3.6% received a CDK4/6 inhibitor, and 3.9% received endocrine therapy only. The overall pCR was 10.71%, with no significant difference between HER2-low and HER2-zero groups [85].

In another study of 61 HR+ patients, the pCR rate in HER2-low was significantly lower than in HER2-zero (7.1% vs. 27.3%) [64]. In a larger study of 769 patients, the pCR rate in HER2-low patients was 18.1%; in HER2-zero patients, it was 30.1% [63]. Another study of 293 HER2-low patients reported an overall pCR rate of 33.8%, with TNBC significantly higher than HR-positive. In patients without pCR, changes in HER2 status were observed in residual disease and were more prevalent in TNBC (21.1% vs. 14.9%) [86]. In the WSG-ADAPT-HR+/HER2 study, the pCR rate in the HER2-low group was significantly lower than in the HER2-zero group. No difference was observed in the efficacy of regimens containing or lacking anthracyclines based on HER2 status. Increased HER2 expression during treatment (change from HER2-zero to HER2-low) was associated with better survival [77]. In molecular subgroups, HER2-low in HR-positive patients is associated with better prognosis (recurrence-free survival and distant metastasis-free survival), whereas no such association is seen in HR-negative patients (TNBC) [44].

Furthermore, changes in HER2 status sometimes occur during treatment. Discordance of HER2 status between primary and metastatic samples or post-treatment creates a major challenge in therapeutic management. One challenge in breast cancer is changes in HER2 status; for instance, 15.2% of patients changed status from HER2-zero to HER2-low [35], and after neoadjuvant chemotherapy, 42.7% converted from HER2-zero to HER2-low [36], making therapeutic decision-making difficult. Additionally, changes in HER2 status between primary and metastatic tumors pose challenges in treatment selection [9,37,38,39]. In the WSG-ADAPT-HR+/HER2 study, re-evaluation of HER2 after 3 weeks of induction endocrine therapy increased the prevalence of HER2-low from 64.6% to 75.2%, and 60.2% of baseline HER2-zero cases converted to HER2-low. In all three evaluations, it was shown that 83.5% of tumors were HER2-low in at least one evaluation [87]. In addition, of 48 primary HER2-low tumors in patients with brain metastases (BrM), 23 remained HER2-low, 15 converted to HER2-zero, and 10 to HER2-positive; additionally, from initial HER2-zero, 13 converted to HER2-low. Furthermore, comparing extracranial metastases with BrM, out of fifteen extracranial HER2-low cases, five cases converted to HER2-zero and three to HER2-positive, and out of fifteen extracranial HER2-zero cases, seven cases converted to HER2-low [39].

### 3.6. Survival Outcomes and Prognosis

#### 3.6.1. Clinical Stage and Survival

In various studies, patients diagnosed with early-stage breast cancer and HER2-low expression had better survival and prognosis compared to the HER2-zero group; however, in most multivariate analyses, survival status was mainly attributed to tumors with positive hormone receptors (HR-positive), and HER2-low status was not an independent factor [88]. In a population of 10,186 patients with HR-positive and HER2-negative breast cancer, HER2-low status was independently associated with improved disease-free survival (DFS), overall survival (OS), and breast-cancer-specific survival (BCSS) [89], and this improvement remained stable after 5 years of follow-up [90]. Similarly, a cohort study in Denmark (adjusted for age, year of diagnosis, tumor size, lymph node status, histological type and grade, estrogen receptor status, local therapy, and intended treatment protocol) showed that HER2-low was associated with better time to recurrence (HR: 0.92, *p* = 0.03) and overall survival (HR: 0.94, *p* = 0.02) after multivariate analysis [91]. Likewise, a cohort study in Brazil (adjusted for clinical stage, chemotherapy, hormone therapy, radiotherapy, pathological complete response, and race) showed that HER2-low was associated with worse survival in the triple-negative subgroup compared with HER2-zero (55.4% vs. 67.7%; *p* < 0.001), with no significant difference in HR-positive patients [92]. Some studies reported no significant difference in DFS and OS between HER2-low and HER2-zero patients [93,94]. It should be noted that the adjusted covariates varied considerably in each study, which prevents direct comparison of reported hazard ratios across different populations.

In the TNBC subgroup, results are somewhat heterogeneous; some studies reported improved BCSS in locally advanced stages (stage III) [95], while others found no significant difference between HER2-low and HER2-zero groups [69,95,96,97,98,99]. Furthermore, in invasive lobular carcinoma (ILC), HER2-low status was associated with worse DFS compared to HER2-zero [100]. In a study in Finland on patients at stage A2 with one to two metastatic lymph nodes, HER2-low led to better overall survival [45]. Moreover, ER-positive patients without extensive metastasis had HER2-low independently associated with improved disease-free survival [101].

In metastatic disease, treatment with T-DXd in HER2-low patients resulted in an objective response rate of 37% and a mean PFS of 11.1 months [102]. However, the efficacy of RC48 in these patients, with a mean PFS of 4.28 months and a disease control rate of 64%, showed less performance compared to HER2-positive patients [79]. In patients with brain metastases, HER2-low was associated with shorter time to brain metastasis but longer overall survival compared to HER2-zero, especially in HR-negative patients [103]. In addition, in recurrent and de novo MBC patients, HER2-low was associated with an 11% and 23% reduction in mortality risk, respectively, compared to HER2-zero [48]. Conversely, in a large population of 14,642 patients, it was shown that HER2-low in the HR-positive subgroup led to slightly worse survival, and this association in the HR-negative subgroup reached the threshold of significance [16]. Furthermore, in another cohort study with a population of over 3376 people, no significant difference was seen in recurrence-free survival and overall survival between the two groups [18].

#### 3.6.2. Factors Affecting Prognosis and Survival in the HER2-Low Subgroup

In HER2-low patients, multiple factors are associated with patient survival. Positive estrogen receptor (ER) and progesterone receptor (PR) status were significantly associated with better survival [79,104,105], while regional lymph node involvement [79] and high proliferation index (Ki-67 > 30%) were associated with poorer outcomes [35,106], and liver metastasis was surprisingly associated with better survival [79]. On the other hand, achieving pCR after neoadjuvant chemotherapy [36] and high infiltration of tumor lymphocytes (TILs) [107] significantly led to improved survival. On the other hand, HER2 instability during disease progression was associated with worse survival after recurrence in patients with a stable HR-positive/HER2-negative phenotype [108].

The prognosis of patients with HER2-low breast cancer is not uniform across all disease stages and for every disease; its effects on survival depend on factors such as hormone receptor status (HR), lymph node involvement, proliferation index (Ki-67), and treatment response. Generally, patient prognosis, besides low HER2 expression, is influenced by various clinical factors (Table 4).

**Figure 1:**
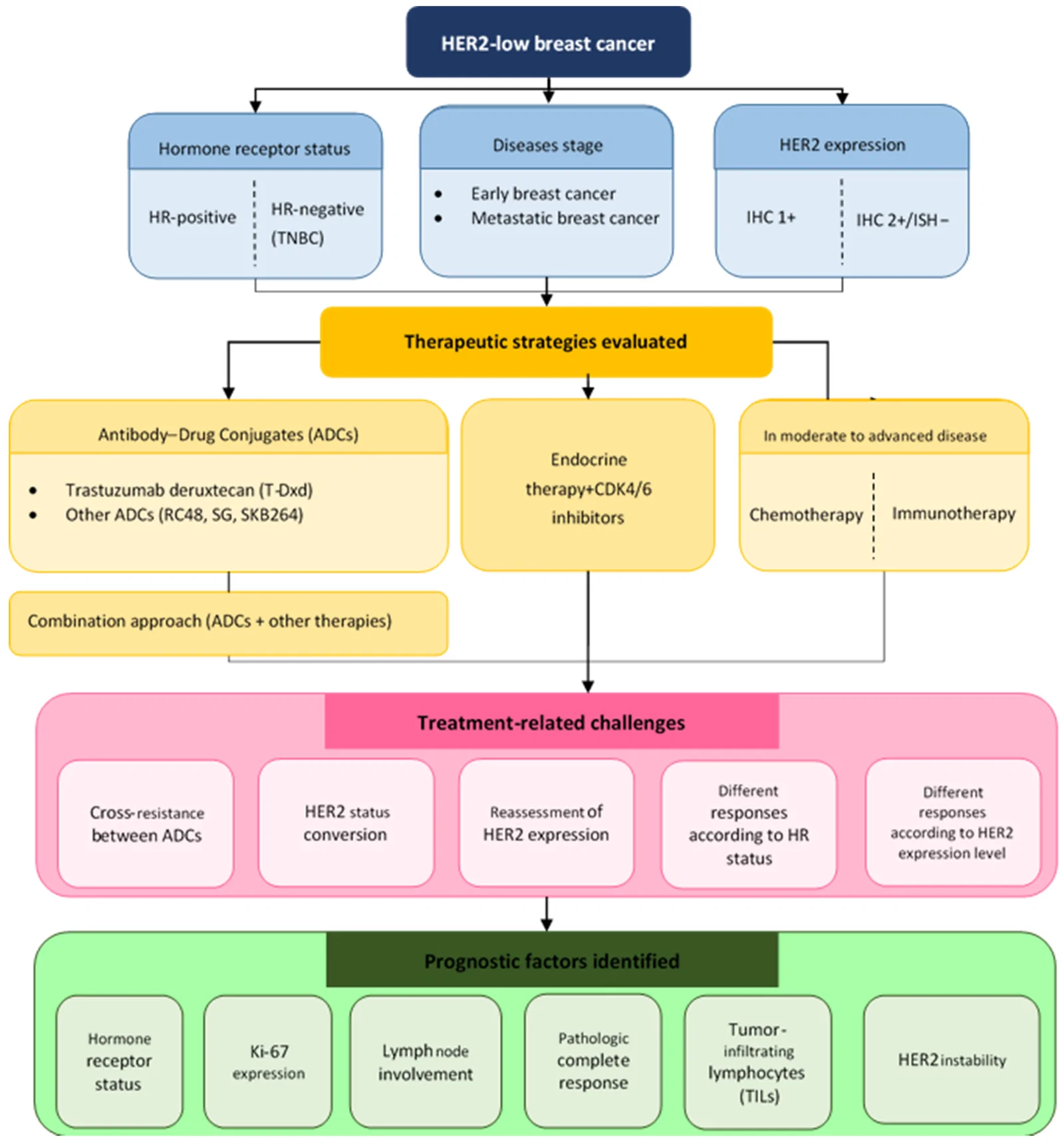
Overview of evidence related to therapeutic strategies, challenges, and prognostic factors in HER2-low breast cancer based on the included studies.

Figure 1 provides an overview of treatment strategies, challenges, and prognostic factors identified in HER2-low breast cancer based on the studies conducted.

## 4. Discussion

This narrative review, synthesizing evidence from 91 studies, investigated the epidemiology, clinicopathological characteristics, diagnostic challenges, imaging features, treatment outcomes, survival, and prognosis of HER2-low tumors. Overall, the findings highlight significant heterogeneity in the reported prevalence, diagnostic classification, treatment response, and prognostic significance of HER2-low tumors [10,11,12,13,14,15,16,17,18,19,20,26,27,45,46,47,59,60,61,62,63,64]. Although there is still no complete consensus among specialists regarding the nature of this subgroup [32,41], its precise diagnosis is of significant importance for the use of therapeutic and drug methods, especially the use of ADCs, which were previously overlooked for this subgroup [26,27,28,29,30,41,77]. Therefore, the clinical significance of low HER2 status appears to depend on the context in which it is assessed, including HR status, disease stage, treatment exposure, and diagnostic methodology [30,85].

The observed prevalence of HER2-low showed a wide range between studies (more than twofold), which may reflect differences in assessment criteria, HER2 scoring, study populations, and other methodological factors [8,9,10,11,12,13,14,15,16,17,18,19]. Similarly, a narrative review showed that this subgroup accounts for approximately 60% of breast cancer cases, and its precise diagnosis and treatment hold significant value [109]. Additionally, another review indicated that half of breast cancer patients have the HER2-low subgroup, and the emergence of ADCs like T-DXd highlights the importance of accurately diagnosing this subgroup [110]. Consistent with previous studies, our review showed that most HER2-low tumors are hormone receptor-positive, a fact confirmed across various populations [111,112,113].

One of the obstacles to selecting optimal targeted therapies is the diagnostic challenge in distinguishing HER2-low from HER2-zero and HER2-ultralow. The difficulty in distinguishing IHC 0 from IHC 1+, rather than reflecting simple observer variability, reflects the biological continuum of HER2 expression and the semi-quantitative nature of current IHC assessment [32,33,34]. This disagreement in diagnosing HER2-low across different laboratories (39.8% to 95%) and in different antibody clones (such as DG44 or 4B5-Ultraview) further suggests that diagnostic variability is not solely related to individual pathologists, but also depends on laboratory and assay-related factors [14]. Emerging technologies such as AI-based analyses, computational pathology, and the use of certain software can promise a reduction in variability among pathologists. However, the reviewed studies showed that consensus regarding the role of AI in diagnosis remains incomplete, and many specialists are uncertain about its differentiation capabilities [34,41]. In a narrative review, using an AI model calibrated based on quantitative immunohistochemistry (qIHC) data, it was possible to continuously detect low and very low levels of HER2, helping to reduce variability among pathologists and standardize diagnosis [114].

One of the most important findings of this review is the intragroup heterogeneity within the HER2-low subgroup. Although this subgroup is defined as IHC scores of 1+ or 2+ with negative FISH/ISH [10,11,13,20,23,24,25,42,45,46,47,54,55,56,57,58], the treatment response in IHC 2+ patients was significantly better than in IHC 1+ patients [27], and a genomic study showed at least three distinct molecular clusters (CS1–CS3) in HER2-low, associated with different responses to chemotherapy, endocrine therapy, and CDK4/6 inhibitors [115]. These findings suggest that classification solely based on IHC may not be beneficial and that there is a need to develop personalized approaches based on individual molecular profiles.

The findings of the present study showed that HER2-low tumors have specific morphological features, including tumor diameter, irregular shape, ill-defined margins, and lower peritumoral edema compared to HER2-positive tumors [47,70,71]. Therapeutically, T-DXd demonstrated a broad efficacy range across various studies; however, the response rate to the first treatment was significantly higher than to the second, and half of the patients showed cross-resistance to ADCs [11,30]. This finding suggests that treatment sequencing may be as important as the selection of a specific ADC, particularly in patients who have previously been exposed to another ADC. Additionally, other treatment methods, including CDK4/6 inhibitors (palbociclib), were associated with a significant increase in PFS in the HER2-low group compared to the HER2-zero group [82]. Despite the high efficacy of T-DXd, the high cost of this drug is considered a challenge in equitable access to treatment. One study showed that treatment costs for HER2-low patients were EUR 12,000, similar to HER2-zero patients and significantly lower than HER2-positive patients [53]. Furthermore, studies in China and the USA showed that the ICER for T-DXd was USD 236,026 and USD 317,494, respectively, against willingness-to-pay thresholds of $35,796 and $150,000, indicating that the price of T-DXd needs to be reduced, and designing cost-effectiveness strategies could be a priority for ensuring equitable patient access to treatment [116,117].

Regarding prognosis, a clear survival advantage associated with low HER2 status was consistently observed in HR-positive patients and in early-stage disease, whereas findings were less consistent in TNBC and other subgroups. This pattern suggests that low HER2 expression alone may not be an independent prognostic determinant and that the observed differences in survival may in part reflect biological and clinical features associated with HR positivity [88]. In a meta-analysis by de Moraes et al. (2025) involving 38 studies with 70,104 patients, it was shown that the HER2-low subgroup had a significantly lower pCR rate compared to HER2-zero, but in multivariate analysis, disease-free survival (HR = 0.83) and overall survival (HR = 0.80) favored the HER2-low subgroup [118]. Consistent with these findings, a meta-analysis with 115,182 patients showed that despite the lower pCR rate in the HER2-low group compared to the HER2-zero group, disease-free Survival and overall survival were higher in the HER2-low subgroup [119]. Overall, the current evidence indicates that HER2-low is a distinct subgroup with unique characteristics and requires differentiated diagnostic and therapeutic approaches.

This narrative review, by synthesizing evidence from various studies, provides a relatively comprehensive overview of the clinical, diagnostic, therapeutic, prognostic, and survival dimensions of HER2-low tumors. However, limitations in interpreting the findings should be kept in mind. The narrative nature of this study, the methodological diversity of included studies, differences in studied populations, and definitions of HER2-low do not allow for a quantitative meta-analysis. Additionally, some included studies are retrospective or clinical trials with subgroup analysis, which may be subject to selection bias. Another limitation of this study is the lack of systematic evaluation of the quality of included studies using standard tools, which could impact the interpretation of findings. Furthermore, variability in antibody clones, reagent sensitivity, and laboratory-specific quality control protocols across different centers represents a significant source of diagnostic heterogeneity that cannot be fully adjusted for in a narrative review. Nevertheless, priority was given to studies with large sample sizes, cohorts, or clinical trials. Despite these limitations, this review, due to the comprehensiveness of sources and coverage of new topics such as AI and radiomics, can provide a clear picture of the current state of knowledge in this field and serve as a basis for designing future research and potentially revising clinical guidelines.

## 5. Conclusions

This narrative review demonstrated that HER2-low tumors constitute a significant portion of breast cancer and possess epidemiological, clinicopathological, imaging, and therapeutic characteristics that distinguish them from other HER2 subgroups. However, multiple challenges exist in managing and treating these patients, including poor agreement among specialists in diagnosis, laboratory variability, intragroup heterogeneity, cross-resistance to treatment, and high drug costs. Furthermore, the prognosis of these patients is influenced by hormone receptor status, proliferation index, lymph node involvement, and treatment response, and HER2-low status alone is not an independent prognostic factor. Therefore, attention to molecular profiling, standardization of diagnostic methods, and the development of cost-effectiveness guidelines appear necessary to improve clinical outcomes and ensure equitable access to treatment for patients with breast cancer.

## Figures and Tables

**Table 1:** Definitions of HER2 based on reviewed studies.

Classification	Definition Stated in Studies	Reference
HER2-positive	IHC 2+ or IHC 3+ with positive FISH/ISH	[13,21,23,41]
HER2-low	IHC 1+ or IHC 2+ with negative FISH/ISH	[10,11,13,20,23,24,25,42,45,46,47,54,55,56,57,58]
HER2-zero	IHC 0	[21,45,47,56,57,58]
HER2-ultralow	IHC 0 with weak membranous staining	[32,41,59]

Definitions based on ASCO/CAP criteria: IHC 1+: faint, incomplete membrane staining in more than 10% of tumor cells; IHC 2+ with negative FISH/ISH: equivocal protein staining, but gene amplification tests show no extra gene copies; traditional testing: classified breast cancer strictly as HER2-positive or HER2-negative; wider spectrum: includes HER2-positive, HER2-low (IHC 1+ or IHC 2+/ISH-negative), HER2-ultra-low (IHC 0 with >0% to ≤10% faint/incomplete staining), and HER2-null (complete absence of staining).

**Table 3:** Imaging features and performance of prediction models in HER2-low tumors.

Category	Feature/Model	HER2-Low	HER2-Zero	HER2-Positive	Key Notes	Reference
MRI Morphological Features	Number of lesions	27%	24%	40%	Significantly higher in HER2-positive compared to the other two groups (*p* < 0.05)	[47,71]
	Morphology (NME)	26%	28.5%	36%	Significantly higher in HER2-positive compared to the other two groups (*p* < 0.05)	
	Peritumoral edema	54%	49%	69%	Significant difference among all three groups (*p* < 0.05)	
	Irregular shape	74.5%	53.7%	-	HER2-low significantly higher than HER2-zero	
	Washout pattern in DCE	77%	81%	88%	Significant difference between HER2-low and HER2-positive (*p* < 0.05)	
	Initial enhancement rate	0.737	0.617	-	Significant difference (*p* < 0.05)	
	Mean ADC (×10^−3^ mm^2^/s)	0.89	0.91	0.94	HER2-low lower than HER2-positive (*p* = 0.043 in multivariate analysis)	
	Minimum ADC (×10^−3^ mm^2^/s)	0.987	1.089	-	Significant difference (*p* < 0.01)	
Radiomics Models	Habitat Model (XGBoost)	-	-	-	AUC between 0.844 and 0.877 across three centers	[8,71,72]
	Combined Nomogram (TIC + Rad-score)	-	-	-	Training AUC: 0.892, Test AUC: 0.886	
	Neural Network Model (Morphology + Initial enhancement rate + ADC)	-	-	-	Training AUC: 0.757, External Validation AUC: 0.791	
Machine Learning Models	Decision Tree (Multifocal lesions, Edema, Margin)	-	-	-	Training AUC: 0.79, Test AUC: 0.69	[47]
	SVM (Multifocal lesions, Edema, Margin)	-	-	-	Training AUC: 0.79, Test AUC: 0.67	
	kNN	-	-	-	Test AUC: 0.64	
	ANN	-	-	-	Test AUC: 0.61	
Novel Biomarkers	ITH Score (Intra- and Peritumoral Heterogeneity) and its impact on prediction models	0.21 ± 0.50 (vs. HER2+) and 0.17 ± 0.71 (vs. HER2-0)	0.19 ± 0.53	0.18 ± 0.32	ITH score was higher in HER2-low than in HER2-zero and lower than in HER2-positive (*p* < 0.001). Adding ITH to models significantly improved predictive performance (NRI: 0.28–0.32, IDI: 0.11–0.14, *p* < 0.001 to *p* = 0.008)	[73]

(-) indicates that the reference study did not report this specific characteristic for that subgroup.

**Table 4:** Prognostic implications of HER2-low status across different clinical subgroups and disease stages.

Clinical Status	Overall Result	Level of Agreement Among Studies	Reference
Early-stage HR+	HER2-low is primarily associated with better survival	High	[88,89,90,91,92,101]
TNBC	Heterogeneous results	Moderate	[69,95,96,97,98,99]
ILC	HER2-low is associated with worse DFS compared to HER2-zero	Low	[100]
Metastatic Disease	HER2-low shows better response to ADCs	High	[11,30,79,102]
Overall Prognosis	HER2 status alone is not an independent prognostic factor and depends on other clinical factors	High	[16,18,45,79,88,101,103,104,105,108]

HR+: hormone receptor-positive; TNBC: triple-negative breast cancer; ILC: invasive lobular carcinoma; DFS: disease-free survival; ADC: antibody–drug conjugate. The level of agreement (high/moderate/low) was qualitatively determined by the authors based on the number and consistency of supporting studies and their sample size.

## Data Availability

No new data were created or analyzed in this study. Data sharing is not applicable to this article.

## Abbreviations

ADC	Antibody–Drug Conjugate
AI	Artificial Intelligence
ANN	Artificial Neural Network
AUC	Area Under the Curve
BCSS	Breast-Cancer-Specific Survival
BI-RADS	Breast Imaging Reporting and Data System
CBR	Clinical Benefit Rate
DCE	Dynamic Contrast-Enhanced
DFS	Disease-Free Survival
ER	Estrogen Receptor
FISH	Fluorescence In Situ Hybridization
HER2	Human Epidermal Growth Factor Receptor 2
HR	Hormone Receptor
IHC	Immunohistochemistry
ILD	Interstitial Lung Disease
ISH	In Situ Hybridization
ITH	Intratumoral and Peritumoral Heterogeneity
KNN	k-Nearest Neighbors
MBC	Metastatic Breast Cancer
MRI	Magnetic Resonance Imaging
NME	Non-Mass Enhancement
ORR	Objective Response Rate
OS	Overall Survival
PCR	Pathological Complete Response
PFS	Progression-Free Survival
PLD	Pegylated Liposomal Doxorubicin
PR	Progesterone Receptor
RC48	Disitamab Vedotin
SG	Sacituzumab Govitecan
STIL	Stromal Tumor-Infiltrating Lymphocytes
SVM	Support Vector Machine
T-DXd	Trastuzumab Deruxtecan
TNBC	Triple-Negative Breast Cancer
XGBoost	Extreme Gradient Boosting
